# Non-exanthematous mpox infections in Nigeria: a possible explanation of the sporadic outbreaks in city centers?

**DOI:** 10.11604/pamj.supp.2025.50.1.46087

**Published:** 2025-04-08

**Authors:** Simeon Cadmus, Matthias Besong, Victor Akinseye, Samuel Ayanwale, Adekunle Ayinmode, Daniel Oluwayelu, Hyacinth Ebenyi, Adeola Fowotade, Michael Olisa, Emeka Sampson, Ephraim Nwanga, Terese Gabriel Orum, Opeyemi Alayande, Rasheed Ansumana, Eniola Cadmus, Solomon Odemuyiwa, Oyewale Tomori

**Affiliations:** 1 Department of Veterinary Public Health and Preventive Medicine, University of Ibadan, Ibadan, Nigeria,; 2 Damien Foundation Genomics and Mycobacteria Research and Training Centre, University of Ibadan, Ibadan, Nigeria,; 3 Centre for Control and Prevention of Zoonoses, Faculty of Veterinary Medicine, University of Ibadan, Ibadan, Nigeria,; 4 Nigerian Institute of Medical Research, Yaba, Lagos, Nigeria,; 5 Department of Chemical Sciences, Augustine University Ilara-Epe, Lagos State, Nigeria,; 6 Department of Veterinary Parasitology and Entomology, University of Ibadan, Ibadan, Oyo State, Nigeria,; 7 Department of Veterinary Microbiology, University of Ibadan, Ibadan, Oyo State, Nigeria,; 8 Department of Public Health and Disease Control, Ebonyi State, Nigeria,; 9 Infectious Disease Institute, College of Medicine, University of Ibadan, Ibadan, Nigeria,; 10 Ebonyi State Ministry of Health, Centenary City, Abakaliki, Ebonyi State, Nigeria,; 11 Department of Veterinary Services, Ministry of Agriculture and Natural Resources, Abakaliki, Ebonyi, Nigeria,; 12 Regional Disease Surveillance System Enhancement Project, Abuja, Nigeria,; 13 School of Community Health Sciences, Njala University, Bo, Sierra Leone,; 14 Department of Community Medicine, College of Medicine, University of Ibadan, Ibadan, Nigeria,; 15 Department of Veterinary Pathobiology, University of Missouri, Columbia, MO, USA,; 16 African Centre of Excellence for Genomics of Infectious Diseases, Redeemer´s University, Ede, Osun State, Nigeria

**Keywords:** Mpox, non-exanthematous, One Health, CAN, Nigeria

## Abstract

**Introduction:**

non-exanthematous presentations in mpox infection are increasingly reported, posing a risk of underestimating the true burden of the disease. In Nigeria, border towns play a unique role in shaping the dynamics of mpox transmission, yet they have received relatively little research attention. This study aims to address this gap by investigating the prevalence of mpox infection in a potential hotspot region of Nigeria.

**Methods:**

a cross-sectional study was conducted during a free medical outreach in Iboko, Izzi Local Government Area, Ebonyi State, Nigeria, from April 15 to May 20, 2024. Participants were educated on the study objectives and provided informed consent for clinical evaluation and sample collection. Blood samples from 75 participants were tested for the mpox virus using quantitative PCR. Socio-demographic data and clinical presentations were also recorded.

**Results:**

the mean age of participants was 51.3 ± 18.0 years, and 73.3% were female. Most participants (78.7%) were farmers, and 52.0% had no formal education. Headache (69.3%) and body pain (37.3%) were the most common clinical symptoms, with only 3.6% presenting with fever. Mpox virus DNA was detected in two participants (2.67%) designated K3 and K4. Both cases lacked exanthematous lesions but reported headache and body pain.

**Conclusion:**

this study identified the presence of non-exanthematous mpox infections in Nigeria, emphasizing the need to recognize and address “subclinical” spreaders to mitigate sporadic outbreaks of mpox infection. In addition, the findings highlight the importance of integrating targeted awareness campaigns to enhance surveillance and improve case detection of asymptomatic mpox in Nigeria.

## Introduction

Human mpox (formerly monkeypox) is an emerging zoonotic disease caused by the monkeypox virus (MPXV). MPXV is an enveloped double-stranded DNA virus belonging to the genus Orthopoxvirus in the family *Poxviridae*. Monkeypox virus infects a taxonomically wide range of mammalian species, but the natural host is unknown [1]. It is one of four Orthopoxvirus species known to cause clinical disease in humans. The virus is considered a high-consequence pathogen that typically causes lesions and clinical symptoms that can be confused with those of smallpox [2-4]. Following the eradication of smallpox, mpox is now regarded as the most important *orthopoxvirus* infection in humans [5]. First identified in a 9-month-old boy in the Democratic Republic of Congo in 1970 [6], mpox is now endemic in 12 countries of Central and West Africa. There are two distinct genetic clades of MPXV: the Congo Basin clade (now renamed as Clade I) and the West African clade (renamed as Clade II) [7-10]. Clade II MPXV isolates are further subdivided into two subclades, IIa and IIb [11-13]. Whereas the Clade I virus causes a smallpox-like illness with a case fatality rate of up to 10% in unvaccinated populations, Clade II viruses cause less severe disease [14,15]. However, the ongoing 2022-2024 multi-country outbreak has been attributed to subclade IIb viruses [16-18].

Mpox is mainly transmitted from animals to humans through direct contact with the blood, bodily fluids, or cutaneous or mucosal lesions of infected animals [9,19]. In most cases, mpox is clinically characterized by a rash and skin eruption, however, a non-exanthematous mpox infection is a form of infection that is not characterized by skin eruption or rash. Different animal species including rope squirrels, tree squirrels, Gambian pouched rats, mice, rabbits, hamsters, porcupines, non-human primates, black-tailed prairie dogs, African brush-tailed porcupines, rats, and shrews are susceptible to MPXV, although the precise natural reservoir(s) are unknown [5,9]. The possibility of human-to-human transmission has been reported [9,20]. There are limited reports of mpox presenting as presymptomatic, asymptomatic, or in individuals experiencing prodromal symptoms without rash. Hence, the contribution of these infections to transmission is unknown. In addition, vertical transmission through the placenta and during childbirth [9,21,22] as well as sexual transmission via intercourse with infected partners, especially bisexual, and men having sex with men (MSM), have been documented [8,23].

In 2022, it was recorded that since the re-emergence of mpox in Nigeria in September 2017, the country recorded one of the highest cases of the disease in Africa [24]. Between 2017 and October 27, 2024, a total of 5259 suspected cases, 1204 (22.9%) confirmed cases, and 17 deaths (CFR=1.4%) have been recorded [25]. In 2024 alone, as of 27 October 2024, 118 confirmed cases have been reported, with males accounting for 74 (63%) of the total confirmed cases. Six high-burden states, including Plateau, Cross River, Bayelsa, Delta, Abia, Lagos, and Akwa-Ibom, have been identified [25]. Although the high-burden states in Nigeria are located within the forest belt of the country [26,27], which is consistent with what is known about mpox transmission in West and Central Africa [28], most confirmed cases reported so far in Nigeria have occurred in urban or peri-urban areas where the likelihood of contact with known mpox reservoirs is minimal [25,29]. In addition, there has been an expansion of human mpox beyond rainforest areas in Nigeria, with confirmed cases reported in the northern dry savannah areas [30]. These findings suggested that the epidemiology and ecology of mpox in Nigeria might be changing. In this study, we employed a Community Action Network (CAN) approach to explore the incidence of active, ongoing MPXV infection in the border town of Izzi, Local Government Area (LGA) of Ebonyi State, Nigeria. The CAN is an initiative set up in strategic geographical regions of Nigeria for reporting syndromes of zoonotic diseases to appropriate human and animal health authorities. Our results confirmed occult circulation of MPXV among patients presenting with syndromes that may not initially suggest mpox. These preliminary findings provide the basis for a wider investigation of MPXV viremia in Nigeria.

## Methods

**Figure 1:**
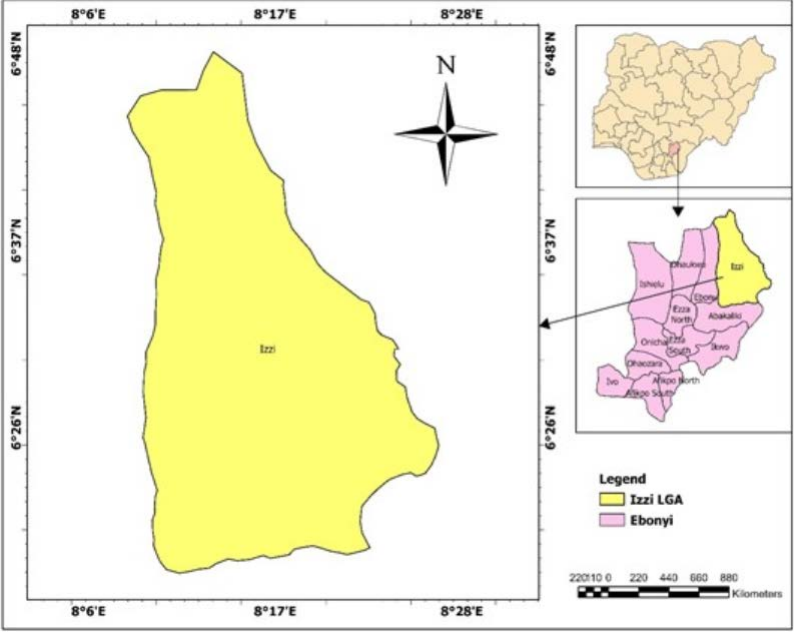
map of Nigeria showing Ebonyi State and Izzi LGA

**Study site:** this study was conducted in the Izzi Local Government Area (LGA) in Ebonyi State, Nigeria. It shares borders with Abakaliki and Ikwo LGAs, as well as parts of Cross River and Benue states. Participants for this study were recruited from Abaja, Igbeagu, Mgbala Ukwu, Ndieze, Ezzinyi Magu, Edukpachi, Ohuruekpe, Okpoduma, and Iboko, which is the LGA headquarters (Figure 1). The majority of the 157,893 residents of Izzi LGA are members of the Izzi sub-ethnic group of the Igbo people. Izzi is 2,264 square kilometers in size, has an average yearly temperature of 27°C, and has an average relative humidity of 70%. The LGA has a significant amount of forest and guinea savannah vegetation. The Izzi people are mainly crop farmers who produce yams, rice, cassava, sweet potatoes, Bambara nuts, and cocoyams. Izzi has a thriving commercial sector with a sizeable food market that attracts customers from Abakaliki, the state capital, adjacent LGAs, and neighbouring states. Hunting and the rearing of domestic animals like pigs and goats are other important sources of income for the Izzi people.

**Study design:** the study involved a cross-sectional design using the One Health approach. A free medical outreach was conducted in Iboko, the Izzi LGA headquarters (Figure 1).

**Medical outreach:** this study was carried out following approval by the University of Ibadan Institutional Research Board (IRB). Participants were first educated about the purpose of the Community Action Network (CAN) activities, followed by a signed consent to obtain samples of body fluids, including blood and urine. Our CAN is a gender-mainstreamed, collectively conscious, community-resilient action group set up in strategic geographical regions of Nigeria to specifically assess clinical syndromes that may be related to zoonotic diseases in animals and humans living in the same community. The main focus of our CAN activities is Lassa fever, Ebola virus disease, COVID-19, and mpox. This study was based on the approach that a free community medical outreach is a viable method of assessing the current health status of a community while generating data to estimate the prevalence of specific diseases in the community. A medical outreach was conducted to give a snapshot of the health status of inhabitants of Izzi LGA at the time of the visit from April 15 to May 20, 2024. Physical examination was carried out to establish a baseline of prevalent disease conditions before commencement of the Community Action Network (CAN) activities. After taking vital signs of participants, blood samples were drawn to screen for malaria using the Rapid Diagnostic Test (careSTART). Retroviral infection (HIV-1/2 AgAb Combo) and hepatitis B virus antigenemia (lateral flow assay for HBsAg) were then assessed.

**Target population:** the entire members of the community (primarily individuals above the age of 18 years) from all 14 wards in Izzi LGA were mobilized for the medical outreach with the support of the LGA authorities. Only individuals who were willing to participate in the free medical outreach and had come to the venue (Local Government Area Health Post, Iboko) voluntarily (including parents and their children) were recruited for the study. Buses were made available by the LGA authorities to transport participants to the venue. One hundred and seventy-four individuals took part in the comprehensive medical screening. However, only 75 gave consent and allowed their blood samples to be collected for testing for the diseases of interest. Importantly, we enrolled individuals without observed or self-reported characteristic mpox rashes.

**DNA extraction:** total nucleic acid was isolated from blood samples according to the manufacturer´s instructions using the Qiagen nucleic acid extraction kit (Qiagen). Briefly, 50 μl of Proteinase K was dispensed into a 1.5 ml Eppendorf tube to which 200 μl of the blood sample was added. After vortexing briefly, 200 μl of lysis buffer was added, and the mixture was vortexed vigorously. The lysate was incubated at 72°C for 10 minutes to complete the lysis process. Afterward, 250 μl of molecular grade 99% ethanol was added to the lysate and vortexed. The lysate-ethanol mixture was pipetted into the center of a spin column and centrifuged at 12,000 revolutions per minute (rpm) for 1 minute to remove cellular components from the spin column. Thereafter, 500 μl of Wash Buffer 1 was dispensed into the center of the spin column and centrifuged at 12,000 rpm for 1 minute. Following this, 500 μl of Wash Buffer 2 was dispensed into the spin column containing the lysate and centrifuged at 12,000 rpm for 1 minute. The spin column was further centrifuged at high speed for 3 minutes to remove excess ethanol. In the final step, 50-60 μl of elution buffer was dispensed into the spin column, and the mixture was incubated for 1 minute at room temperature. Following centrifugation at 12,000 rpm for 1 minute, the eluted nucleic acid was collected in an Eppendorf tube and stored at -80°C until used for PCR.

**Quantitative PCR (qPCR):** a quantitative PCR (qPCR) was performed to amplify the F3L and N3R genes of the MPXV (Annex 1). This was performed using the Luna Universal Probe qPCR Master Mix (New England Biolabs) following the manufacturer’s protocol. The master mix and other reaction components (Annex 1) were thawed at room temperature and then placed on ice. After thawing completely, each component was briefly mixed by gentle vortexing. The total volume for the appropriate number of reactions was determined and used to prepare the assay mix containing all reaction components accordingly. The prepared assay mix was then vortexed gently and aliquoted into PCR tubes. Afterward, the DNA templates were added to each tube. The tubes were capped and spun briefly for 1 minute at 2,500-3,000 rpm. The qPCR reactions were set up in a thermal cycler (Eppendorf, Hamburg, Germany) using the following cycling conditions: initial denaturation at 95°C for 1 min, followed by 40 cycles of denaturation at 95°C for 15 seconds and extension at 60°C for 30 seconds. The annealing temperature was 95°C for 15 seconds. The protocol was validated using a panel of known positive and negative mpox samples.

## Results

**Demographic characteristics of participants:** the population that was screened consisted of 75 participants, the majority (n=55, 73.3%) of whom were females. The mean age of the participants was 51.3 ± 18.0 SD years (Table 1).

**Table 1:** socio-demographic characteristics of sampled participants (n=75)

Variable	Frequency	(%)
**Age group**		
≤ 20 yrs	3	(4.0%)
21-30 yrs	3	(4.0%)
31-40 yrs	20	(26.7%)
41-50 yrs	17	(22.7%)
51-60 yrs	9	(12.0%)
> 60 yrs	23	(30.7%)
**Gender**		
Female	55	(73.3%)
Male	20	(26.7%)
**Level of education**		
No formal education	39	(52.0%)
Primary	21	(28.0%)
Secondary	13	(17.3%)
Tertiary	2	(2.6%)
**Occupation**		
Farmer	59	(78.7%)
Trader	5	(6.7%)
Civil servant	5	(6.7%)
Student	4	(5.3%)
Teacher	2	(2.7%)

**Occupation and level of education of Izzi inhabitants:** most (n=59, 78.7%) of the participants were involved in farming, while others were engaged in other occupations such as trading (n=5, 6.7%), civil service (n=5, 6.7%), public service (1.1%), students (5.3%), and teaching 1 (1.3%). Further, the results showed that 39 (52.0%) of the participants had no formal education, 21 (28.0%) had primary education, 13 (17.3%) had secondary education, and 2 (2.7%) had tertiary education (Table 1).

**Figure 2:**
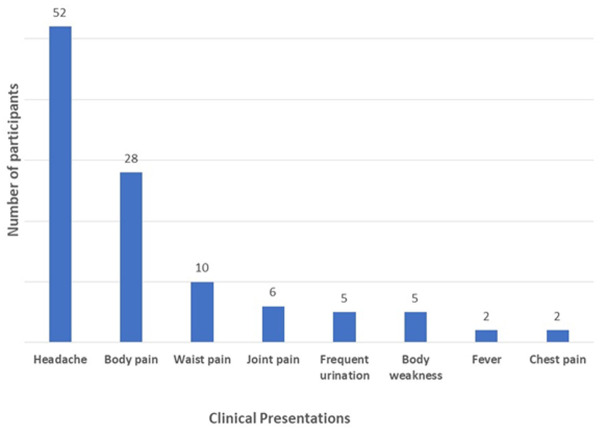
clinical presentation of participants

**Clinical presentation of participants:** the most common clinical presentation recorded was headache (n=52, 69.3%). This was followed by body pain (n=28, 37.3%) and waist pain (n=10, 13.3%). Only 2 (3.6%) of the participants presented with fever (Figure 2).

**Quantitative PCR (qPCR):** two (2.67%) of the 75 participants were positive for MPXV by qPCR. The average Ct values for their blood specimens were 32.00 and 37.00, respectively.

**Socio-demographic and clinical details of MPXV-positive cases:** the laboratory-confirmed cases were designated K3 and K4. K3 was a 7-year-old primary school pupil who resided in Nduofia community in Ndiebor Ishiagu ward of Izzi LGA. K4 was a 40-year-old female farmer from the Ndunwamini community, Igbuhu ward of Izzi LGA. Both cases presented with headache and body pain with no exanthematous lesions. However, K3 was positive for malaria (rapid diagnostic test), indicating co-infection of malaria and mpox. K3 and K4 had no familial relationship and no evidence of physical contact at any time before they participated in the medical outreach (Table 2).

**Table 2:** socio-demographic and clinical details of positive cases (n=2)

ID	Sex	Age (yrs)	Occupation	Clinical presentation		Malaria RDT	Mpox
K3	Male	7	Student	Headache	Body pain	Positive	Positive
K4	Female	40	Farmer	Headache	Body pain	Negative	Positive

## Discussion

The recent reemergence of MPXV infections in Nigeria [29,31] calls for renewed surveillance efforts to facilitate early detection, rapid response and effective treatment of diagnosed cases. In the present study, a medical outreach to a rural community in Ebonyi State, southeast Nigeria, yielded two MPXV-positive cases. The gender distribution of the participants in the present study provides important information for understanding the representativeness of the sample population. The larger proportion of female participants in the study area suggests a higher level of health consciousness among women in the area than men. This is consistent with the findings of [32] who reported that males had poorer health-seeking behavior than females. In addition, the average age of 51 years indicates that most of the participants were older adults. This is a clear pointer to the challenges of rural-urban migration, which is well documented in the southern region of Nigeria [33]. This aged population is more susceptible to infectious diseases due to weakened immune systems. Additionally, their bodies are less able to fight infection due to conditions such as heart disease, lung and kidney infections, and diabetes [34]. Also, the middle-aged adult population serves as a bridge population, with the potential of transmitting infections to both the younger and older populations [35].

Individuals experiencing prodromal symptoms without characteristic mpox rash constitute a significant public health concern, and various factors have been implicated in its occurrence [36]. The clinical parameters of the two MPXV-positive individuals indicate that both had non-exanthematous mpox infection, as they did not present any characteristic skin lesions of the disease. Mpox usually presents with a spectrum of clinical symptoms, including and not limited to skin rash (which is always classical), fever, headache, malaise, pruritus, cough, and conjunctivitis [9]. Also, the age and gender of the two affected individuals show that the occurrence of MPXV infection is without prejudice to age or gender. This is consistent with previous reports of MPXV infections in individuals of different ages and genders [29,30,37]. In addition, the results of this study highlight the need for further research as well as routine testing to better understand the epidemiology of the disease in Nigeria and develop effective strategies for preventing future outbreaks while ensuring the health and well-being of communities worldwide. Farming as an occupation brings people in proximity to wild animals such as squirrels and rabbits that have been implicated as possible reservoirs of MPXV [5]. This suggests that the people of Izzi LGA, who engage primarily in farming, may be at high risk of exposure to the virus, due to the increased possibility of encountering a potential source of the virus in their daily activities. Moreover, previous studies have shown proximity to wild animals as a risk factor for contracting the disease [38,39]. Therefore, further research should be carried out to understand the relationship between farming, which is the main occupation of rural dwellers, and the occurrence of mpox infection. Knowledge acquired from such research can be leveraged for the development of more effective curtailment strategies.

The generally poor education status of the members of the Izzi community (78.7% below secondary school level) may have implications for the prevention and control of infectious diseases. Indeed, lack of formal education has been reported to be a possible indicator of inadequate knowledge of the preventive measures against the disease [40,41]. Moreover, a lack of formal education could increase the likelihood of false beliefs and misconceptions, such as the notion that certain diseases are exclusive to the wealthy and affluent in society, leading to complacency in adopting preventive measures [42]. The detection of MPXV DNA in non-exanthematous individuals in this study is of epidemiological importance. According to a study [43], asymptomatic mpox carriers have the potential for asymptomatic transmission. This category of patients typically continues to engage in their normal daily activities without any precautions and may inadvertently transmit the disease to unsuspecting persons with whom they come in contact [43]. This ongoing, stealthy spread of the virus makes it difficult to trace the infection within communities, which complicates the implementation of control measures. Furthermore, the presence of asymptomatic mpox patients indicates the likelihood of underestimating the true burden of the disease [44]. In addition, since they do not exhibit symptoms of illness, asymptomatic patients typically do not seek medical assistance promptly, thus prolonging the time they remain infectious to others and significantly contributing to the persistence of the disease in the community.

## Conclusion

Based on our findings, there is a possibility of sub-clinical MPXV transmission in Izzi LGA of Ebonyi State, which may also be occurring in other communities in Nigeria. We suspect that these asymptomatic or non-exanthematous cases may play critical roles in the recent sporadic outbreaks of mpox in different cities in Nigeria. Notably, only 75 participants consented to be screened for this study; considering that Izzi LGA has a population of over 122,000, our finding indicates the possibility of more cases being detected if a larger-scale screening for mpox infections had been done.

### What is known about this topic

- Mpox is typically characterized by skin rashes or exanthematous lesions, with clinical manifestations ranging from mild to severe; while the disease has been well-documented in endemic regions, non-exanthematous or atypical presentations have only recently begun to be reported.

### What this study adds

- In this study, we provided evidence for non-exanthematous mpox infections in a rural Nigerian setting, specifically among a population with high zoonotic exposure risks;
- We integrated clinical, diagnostic, and socio-demographic data to identify non-exanthematous mpox cases that might otherwise go undetected;
- This detection of MPXV DNA in asymptomatic individuals without typical rashes emphasizes the need to strengthen surveillance systems and improve case detection to curb transmission of mpox infection in Nigeria.
